# Insertion of a xylanase in xylose binding protein results in a xylose-stimulated xylanase

**DOI:** 10.1186/s13068-015-0293-0

**Published:** 2015-08-15

**Authors:** Lucas Ferreira Ribeiro, Nathan Nicholes, Jennifer Tullman, Liliane Fraga Costa Ribeiro, Carlos Alessandro Fuzo, Davi Serradella Vieira, Gilvan Pessoa Furtado, Marc Ostermeier, Richard John Ward

**Affiliations:** Johns Hopkins University, Baltimore, MD USA; Departamento de Bioquímica e Imunologia, FMRP, Universidade de São Paulo-USP, Ribeirão Preto, SP Brazil; University of Maryland Baltimore County-UMBC, Baltimore, MD USA; Institute for Bioscience and Biotechnology Research, Rockville, MD USA; Universidade Federal do Rio Grande do Norte, Natal, Brazil; Brazilian Bioethanol Science and Technology Laboratory CTBE/CNPEM, Campinas, Brazil; Departamento de Química, Faculdade de Filosofia, Ciências e Letras de Ribeirão Preto, Universidade de São Paulo-USP, Av. Bandeirantes, 3900, Ribeirão Preto, SP 14040-901 Brazil

**Keywords:** Enzyme engineering, Heterotropic allosteric regulation, Non-homologous genes, Semi-rational design

## Abstract

**Background:**

Product inhibition can reduce catalytic performance of enzymes used for biofuel production. Different mechanisms can cause this inhibition and, in most cases, the use of classical enzymology approach is not sufficient to overcome this problem. Here we have used a semi-rational protein fusion strategy to create a product-stimulated enzyme.

**Results:**

A semi-rational protein fusion strategy was used to create a protein fusion library where the *Bacillus subtilis* GH11 xylanase A (XynA) was inserted at 144 surface positions of the *Escherichia coli* xylose binding protein (XBP). Two XynA insertions at XBP positions 209 ([209]XBP-Xyn-XBP) and 262 ([262]XBP-Xyn-XBP) showed a 20% increased xylanolytic activity in the presence of xylose, conditions where native XynA is inhibited. Random linkers of 1-4 Gly/Ala residues were inserted at the XynA N- and C-termini in the [209]XBP and [262]XBP, and the chimeras 2091A and 2621B were isolated, showing a twofold increased xylanolytic activity in the presence of xylose and *k*_cat_ values of 200 and 240 s^−1^ in the 2091A and 2621B, respectively, as compared to 70 s^−1^ in the native XynA. The xylose affinity of the XBP was unchanged in the chimeras, showing that the ~3- to 3.5-fold stimulation of catalytic efficiency by xylose was the result of allosteric coupling between the XBP and XynA domains. Molecular dynamics simulations of the chimeras suggested conformation alterations in the XynA on xylose binding to the XBP resulted in exposure of the catalytic cavity and increased mobility of catalytic site residues as compared to the native XynA.

**Conclusions:**

These results are the first report of engineered glycosyl hydrolase showing allosteric product stimulation and suggest that the strategy may be more widely employed to overcome enzyme product inhibition and to improve catalytic performance.

**Electronic supplementary material:**

The online version of this article (doi:10.1186/s13068-015-0293-0) contains supplementary material, which is available to authorized users.

**Graphical abstract:**
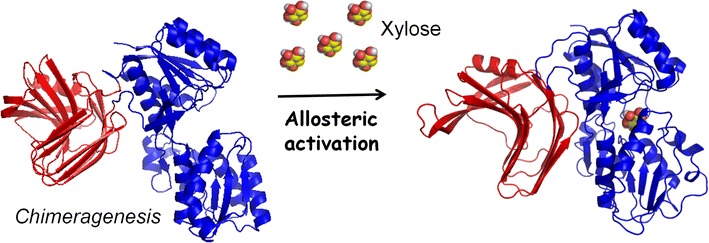
Protein fusion of a GH11 xylanase (in *red*) and a xylose binding protein (XBP, in *blue*) results in a xylanase-XBP chimera that presents allosteric activation of the xylanase activity by xylose (shown as a space-filled molecule bound to the xylanase-XBP chimera)

## Background

The effective production of fermentable hydrolysates from biomass is one of the primary requirements for the production of biofuels and other sustainable products from lignocellulosic material [1]. To reduce water consumption and the costs of distillation equipment, hydrolysis of lignocellulosic material must be conducted at a high concentration of solids [2]. This will inevitably generate high concentrations of the final reaction products, resulting in inhibition of various enzymes involved in the biomass degradation [3, 4] (Additional file 1: Fig. S1a). This inhibition can occur through different mechanisms and in most cases the application of classical enzymology tools is not sufficient to circumvent this problem [4]. Therefore, decreasing product inhibition is a major challenge facing both industrial process development as well as enzyme engineering.

The modification of enzyme properties to either overcome product inhibition or to introduce stimulatory effects by the final products of industrial processes is a significant obstacle that may be overcome by bioengineering [5–7]. Allosterically regulated enzymes present spatially distinct locations for regulation and catalysis and frequently present oligomeric states in which tertiary and quaternary structure changes transmitted across protein–protein interfaces can mediate the communication between allosteric effector binding and the modulation of catalytic activity. These attributes can be exploited by protein engineering strategies that aim to introduce fine modulation of catalytic activity without modification of the active site. A powerful method for constructing allosteric proteins is through random domain insertion, which is potentially a general strategy for introducing coupling between fused domains [8–10].

The endo-β-1,4-xylanase is a key enzyme for biomass saccharification by virtue of its hydrolytic activity against internal β-1,4 glycosidic bonds in the primary chain of xylans [11], polysaccharides that account for approximately one-third of all vegetal biomass on Earth [12]. During the industrial hydrolysis process by a xylanase of the family 11 (XynA) the concentrations of the final products can reach up to ~50% xylobiose and ~10% xylose [13]. Evidence from recent studies shows that inhibition of the xylanase by its final products may limit lignocellulose hydrolysis under conditions of high-solids concentration [14–17].

The xylose liberated by the hydrolysis of xylan by xylanolytic complexes can be captured by the bacteria *Escherichia coli* through the ABC (ATP-binding cassette) type transporter proteins XylF, XylG and XylH [18]. The XylF protein, also known as XBP (xylose binding protein), is a periplasmic sugar binding protein with a high affinity for d-xylose [19]. XBP consists of two similar globular domains that are connected by a flexible hinge region, where the d-xylose binding site is situated at the interface between the two domains. The XBP may adopt at least two different conformations: a ligand-free open form, and a closed ligand bound form. These conformations are interconverted through a relatively large bending movement around the hinge region [20, 21]. This d-xylose sensitive conformational change in the XBP is a fundamental property that may be exploited for the transduction of the input signal (increasing concentration of d-xylose) to an increased xylan hydrolytic activity by the XynA by means of a fusion between the two proteins [22].

This strategy represents a novel platform to engineer enzymes for stimulation by the final product of a specific biotechnological process. The principal goal of this strategy is to develop allosteric enzymes that couple recognition of the final product with increased enzymatic activity (Additional file 1: Fig. S1b). This coupling may arise from inter-domain interactions created after the fusion between the two proteins, and in the present study, the viability of this strategy was demonstrated by combining two unrelated proteins with independent functions. Domain insertion libraries were created, in which the xylanase from glycosyl hydrolase family 11 from *Bacillus subtilis* (XynA) was fused in a semi-rational manner to the xylose binding protein (XBP) from *Escherichia coli* K12. Selected chimeric enzymes showed a positive allosteric modulation by the final product of the process of xylan hydrolysis, d-xylose, and is the first application of the concept of random domain insertion for the engineering of an enzyme that is stimulated by the final product in the degradation of lignocellulose.

## Results

### Semi-rational insertion library construction and screening for xylose-stimulation

Initially, 144 positions in XBP were defined as targets for insertion of XynA. These positions were determined based on previous studies with bifunctional proteins created by the fusion of a catalytic β-lactamase domain with the homologous ribose binding proteins (RBP, 29% identity with XBP) and Glucose Binding Protein (GBP, 21% identity with XBP) [23]. The insertions were performed using the multiplex inverse PCR technique [24] at specific surface positions in the XBP (Additional file 1: Fig. S2).

**Fig. 1:**
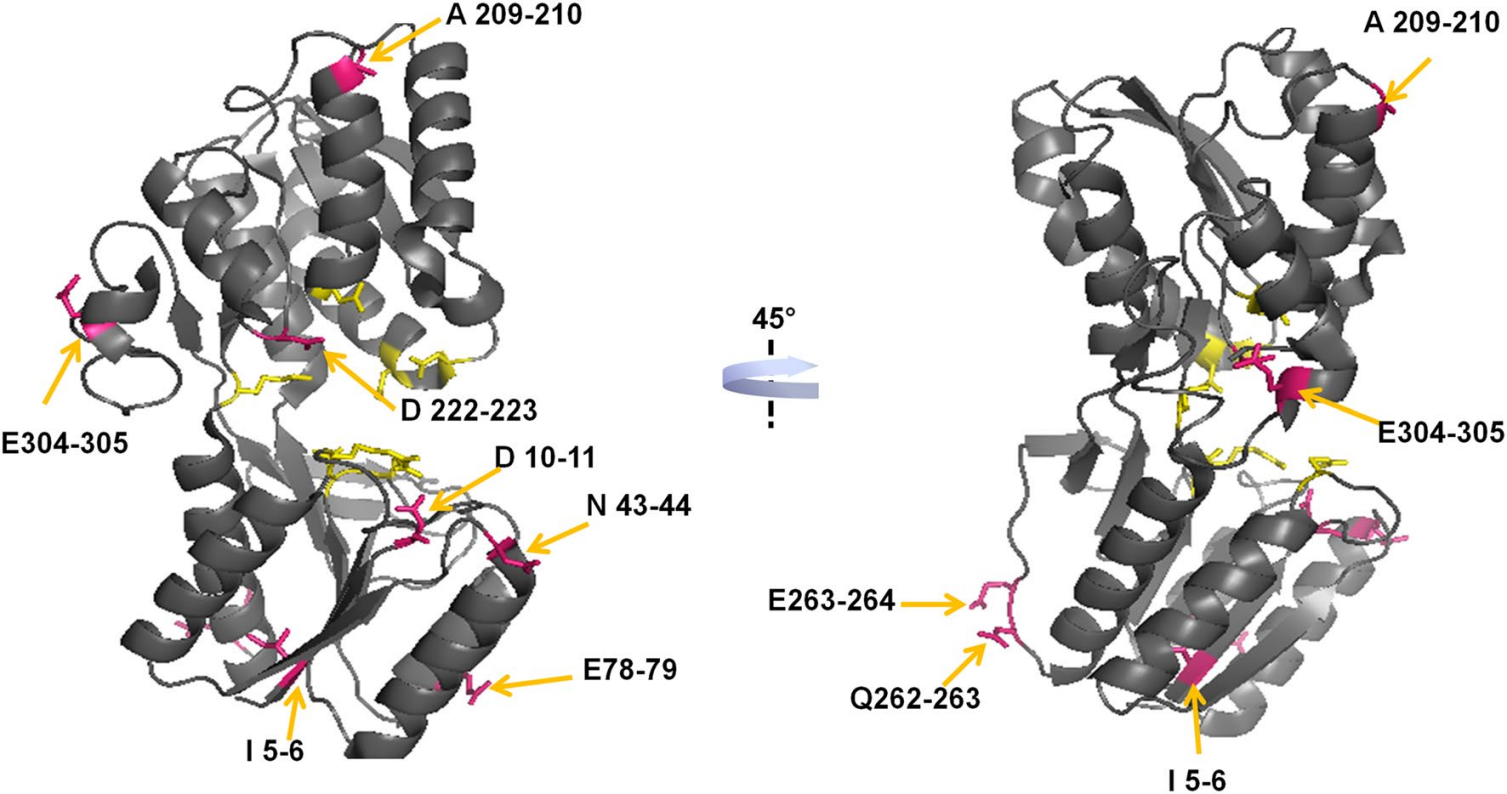
Mapping of clones with positive modulation in the insertion library. Structural representation of XBP showing the insertion sites of the xylanase. The *orange arrows* indicate the insertion points of the xylanase (shown in *pink*). The residues responsible for binding xylose are represented in *yellow*. The structure to the *right* represents a rotation of the structure by +45° around the *y-axis*. The structural representations were prepared using the PyMol software [82].

A total of 2304 clones from the resulting library were analyzed, of which approximately 10% showed clear halos around colonies grown on solid agar containing xylan and xylose. These clones (denominated as XynA+) were inoculated into 384 well plates and stored at −80°C for further analysis. After this initial selection, a second stage of screening was performed to identify which of the XynA+ clones were positively modulated by xylose. A total of 225 XynA+ clones were selected for analysis of xylanase activity in culture supernatants both in the presence and absence of xylose. Of these, 69% (155 clones) showed lower activity in the presence of xylose and 4% (10 clones) showed an activity greater than 10% of the wild-type enzyme in the presence of xylose (Additional file 1: Fig. S3). The clones that showed an increase in xylanase activity of at least 10% in the presence of xylose were submitted to nucleotide sequencing (see Table 1; Fig. 1). The parental XynA shows a 20% decrease in catalytic activity in the presence of xylose; therefore, the increase in activity in these fusion proteins is significant and indicates the elimination of xylose inhibition. It is observed that the insertions were distributed throughout the sequence of XBP (Fig. 1), and the selected clones with the highest activation effect showed an increase in XynA catalytic activity of approximately 20% in the presence of xylose (Table 1). The insertions occurred in the N-terminal domain of XBP, between the end of the helix and the beginning of a loop (A209-210), and also in a loop in the C-terminal domain of XBP (Q262-263) (Fig. 1). Two of the clones have XynA inserted in the same loop of XBP, at positions 262 and 263, and this loop may be a hot spot for bifunctional XBP/XynA proteins.

**Table 1:** Insertion sites of allosteric clones selected from the semi-rational insertion library

Clone	Position	+xyl/–xyl ratio^a^
XynA parental	–	0.80 ± 0.04
1	Q262-263	1.20 ± 0.02
2	A209-210	1.19 ± 0.02
3	E304- 305	1.16 ± 0.01
4	E263-264	1.15 ± 0.02
5	N43-44	1.14 ± 0.02
6	D10-11	1.13 ± 0.01
7	E78-79	1.12 ± 0.02
8	I5-6	1.11 ± 0.02
9	D222-223	1.11 ± 0.03

^a^Xylanase activity in the supernatant (with xylose)/(without xylose). Data represent the mean ± SD of 3 repetitions (*n* = 3).

### Construction and screening of polypeptide linker libraries

Since the greatest increase with xylose stimulation was a modest 20% (see Table 1), two new libraries were created from the selected variants A209-210 and D262-263. These libraries aimed to vary the distance and relative orientation between the XynA and XBP, which we reasoned might modulate the communication between the domains [25]. The length of the linkers was varied between 0 and 4 glycine and/or alanine residues (see Additional file 1: Fig. S4). The linker libraries were screened by measuring the stimulation of xylanase activity on addition of xylose. A total of 2112 clones from the pSkunk2_209 library and 1,344 clones from the pSkunk2_262 library were analyzed. Approximately 1.3% (28) of the clones from the pSkunk2_209 library and 5% (67 clones) from the pSkunk2_262 library showed clear halos on solid agar plates with xylose when compared to plates without xylose (see Additional file 1: Fig. S5a). The clones that showed xylose stimulation were selected and inoculated into deep well plates for analysis of the xylanase activity in the supernatant. Of the clones analyzed from the pSkunk2_209 library, only one showed an increase in the activity of the supernatant above 20% in the presence of xylose (see Additional file 1: Fig. S5b; Table 2). Of the 67 clones selected from the pSkunk2_262 library, 7 showed activity greater than 20% (see Additional file 1: Fig. S5c; Table 2).

**Table 2:** Length and composition of the linkers between the N- and C- termini of xylanase and XBP in the clones presenting the greatest increase in the xylose stimulation effect

Clone^a^	Position	N-ter	C-ter	+xyl/−xyl ratio^b^
1B	262	GGGG	GA	2.22 ± 0.07
2B	262	–	GGG	1.50 ± 0.04
3B	262	GAG	GGG	1.42 ± 0.04
1A	209	AG	GGA	1.38 ± 0.03
4B	262	AA	GA	1.33 ± 0.03
5B	262	GG	GAG	1.30 ± 0.03
6B	262	GGA	A	1.28 ± 0.03
7B	262	AAA	GAA	1.23 ± 0.04

^a^The clone numbers refer to those shown in Additional file 1: Fig. S5b, c.

^b^Ratio of xylanase activity in culture supernatants (xylose present)/(without xylose). Data represent the mean ± SD of three repetitions (*n* = 3).

The two selected variants from the linker libraries that presented the greatest increases were clone 1A (position A209-210; activity ratio without linker = 1.19; with linker = 1.38, hereafter denominated as 2091A) and clone 1B (position Q262-263; activity ratio without linker = 1.20; activity ratio with linker = 2.22, hereafter denominated as 2621B) (Tables 1, 2). This improvement is likely to be a consequence of the increase in the communication between the two domains resulting from the modulation of the interdomain contacts. For position 262, both the length and the composition (clones 3 and 7) of the linkers affected the modulation of the activity by xylose, showing differences of up to 1.8 times in the activation effect between the variants 1 and 7 (position 262). The two clones 2091A and 2621B were selected for further characterization.

### Biochemical characterization

**Fig. 2:**
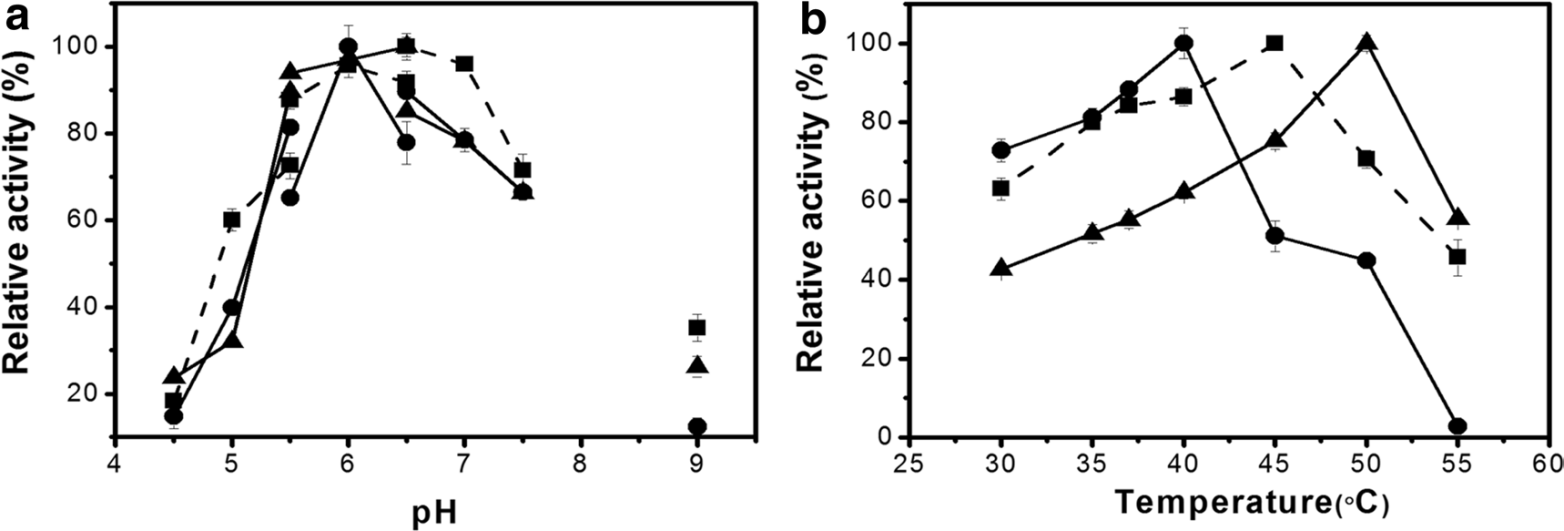
Effects of reaction conditions on xylanase catalytic activity. **a** The effect of pH. The interconnected points represent the following buffers at a final concentration of 50 mM: acetate (pH 4.5–5.5); phosphate (5.5–6.5), MOPS (pH 6.5–7.5) and arginine-NaOH (pH 9.0). **b** The effect of temperature. The *symbols* in both graphs are *filled square*, parental XynA; *filled circle*, 2091A chimera and *filled triangle* 2621B chimera.

The parental proteins XynA and XBP together with the chimeras 2091A and 2621B were expressed in *Escherichia coli* Rosetta™ (DE3) cells, and after purification were subjected to biochemical and kinetic characterization. As shown in Fig. 2a, the optimal pH for the hydrolysis of RBB-xylan was 6.5 for both XynA (in MOPS buffer) and 2621B (in phosphate buffer). The MOPS buffer resulted in a decrease of 15% in the activity of the chimeric enzyme at this pH. The 2091A chimera showed maximum activity at pH 6.0 (phosphate buffer). Both chimeras, together with the parental xylanase, showed activity above 60% over the pH range 5.5–7.5. In pH 5.5 acetate buffer the chimeric enzymes showed greater activity relative to the parental xylanase (XynA; 72 ± 3%; 2091A; 81 ± 2% and 2621B; 90 ± 2%). The 2091A showed lower activity at pH 9.0 (10%) when compared to XynA (40%) and 2621B (26%).

The effect of temperature on the catalytic activity of both chimeric enzymes and the parental xylanase was evaluated at pH 5.5 in acetate buffer. The chimeric enzymes showed a displacement in the maximum activity as a function of temperature relative to XynA, which showed a maximum at 45°C, where 2091A and 2621B showed maximum activity at 40 and 50°C, respectively (Fig. 2b).

### Kinetic characterization

Since the majority of glycosyl hydrolases have an optimal pH around 5.5 and biomass hydrolysis processes involving microorganisms preferentially occur between 30 and 37°C [26–29], the kinetic parameters were determined at pH 5.5 at 37°C (Table 3). The values for the catalytic efficiency (*k*_cat_/*K*_M_) of XynA in the absence and presence of xylose were essentially the same; however, a ~10% decrease was observed in the *k*_cat_ value in the presence of xylose. Additionally, differences between the parental xylanase and the xylanase activity in the chimeric enzymes were observed (Table 3). The largest difference was between the values of the *k*_cat_, which in the absence of xylose was ~2 times greater in the chimeric enzymes than that observed for the parental XynA. In the presence of xylose, this increase was even more pronounced, reaching ~3 times for 2091A and ~3.5 times for 2621B. The xylose activation effect for the pure enzymes (ratio of *k*_cat_ (+xylose)/*k*_cat_ (−xylose)) was 1.33 for 2091A and 1.71 for 2621B, similar to the values obtained during screening with the crude extract (Table 2). The catalytic efficiency in the presence of xylose of the hybrid enzymes was ~2.5 times greater than the value observed for the parental xylanase under the same conditions.

**Table 3:** Kinetic parameters of chimeric enzymes compared with parental enzymes

	Parental XynA		2091A		2621B	
	−Xylose	+Xylose	−Xylose	+Xylose	−Xylose	+Xylose
*K*_M_^a^	1.5 ± 0.1	1.6 ± 0.1	1.7 ± 0.2	1.8 ± 0.1	2.1 ± 0.1	2.4 ± 0.2
*k*_cat_^b^	78 ± 2	70 ± 3	150 ± 8	200 ± 7	140 ± 5	240 ± 8
*k*_cat_/*K*_M_	52 ± 3	44 ± 1	89 ± 8	111 ± 3	67 ± 1	100 ± 7

Data represent the mean ± SD of two independent preparations.

^a^mg mL^−1^.

^b^s^−1^.

### Ligand-affinity measurements

Alterations in the intrinsic tryptophan fluorescence emission were used to monitor conformation changes on d-xylose binding to XBP and the chimeric enzymes. Sugar binding caused an increase in fluorescence intensity of 30% in XBP and a decrease of 15% in the chimeras. The *K*_d_ values estimated from these data were 0.15 ± 0.02, 0.16 ± 0.01 and 0.14 ± 0.03 µM for 2091A, 2621B and parental XBP respectively, demonstrating that all three proteins had a similar affinity for xylose.

### Enzyme activity against milled sugarcane bagasse

**Fig. 3:**
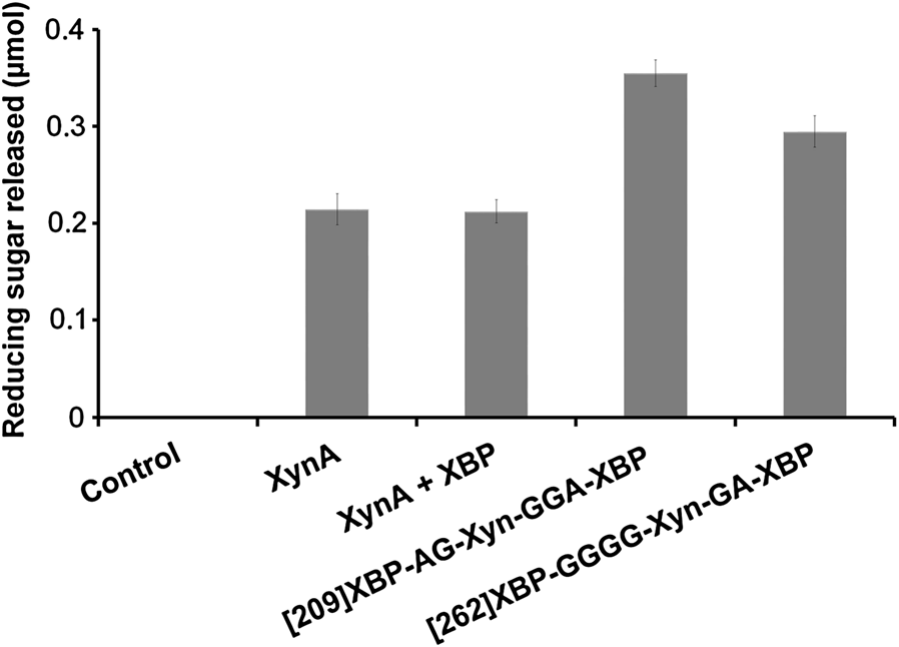
Enzyme activity against milled sugarcane bagasse. The bagasse was treated with equal molar concentrations of the parental xylanase (XynA), or a mixture of XynA and XBP (XynA + XBP), or the chimeras (2091A or 2621B). The control was treated under the same conditions but without enzyme. The result shows total reducing sugar release in micromoles. Triplicate assays were performed in 50 mM phosphate buffer (pH 6.0) at 37°C for 2 h (see experimental procedures for further details).

The effect of the chimeric enzymes against a natural lignocellulose substrate was evaluated by measuring the total reducing sugar released after treatment of milled sugarcane bagasse with the parental enzyme (XynA), with an equimolar mixture of the two proteins (XynA + XBP), or with the chimeras 2091A and 2621B (Fig. 3). The effect of XynA alone was essentially the same as a mixture of the XynA + XBP, demonstrating that XynA activity accounted for all reducing sugar release. The amount of reducing sugar release from bagasse by the 2091A and 2621B chimeras was 67 and 40% greater than the parental enzyme, respectively.

### Molecular dynamics simulations

**Fig. 4:**
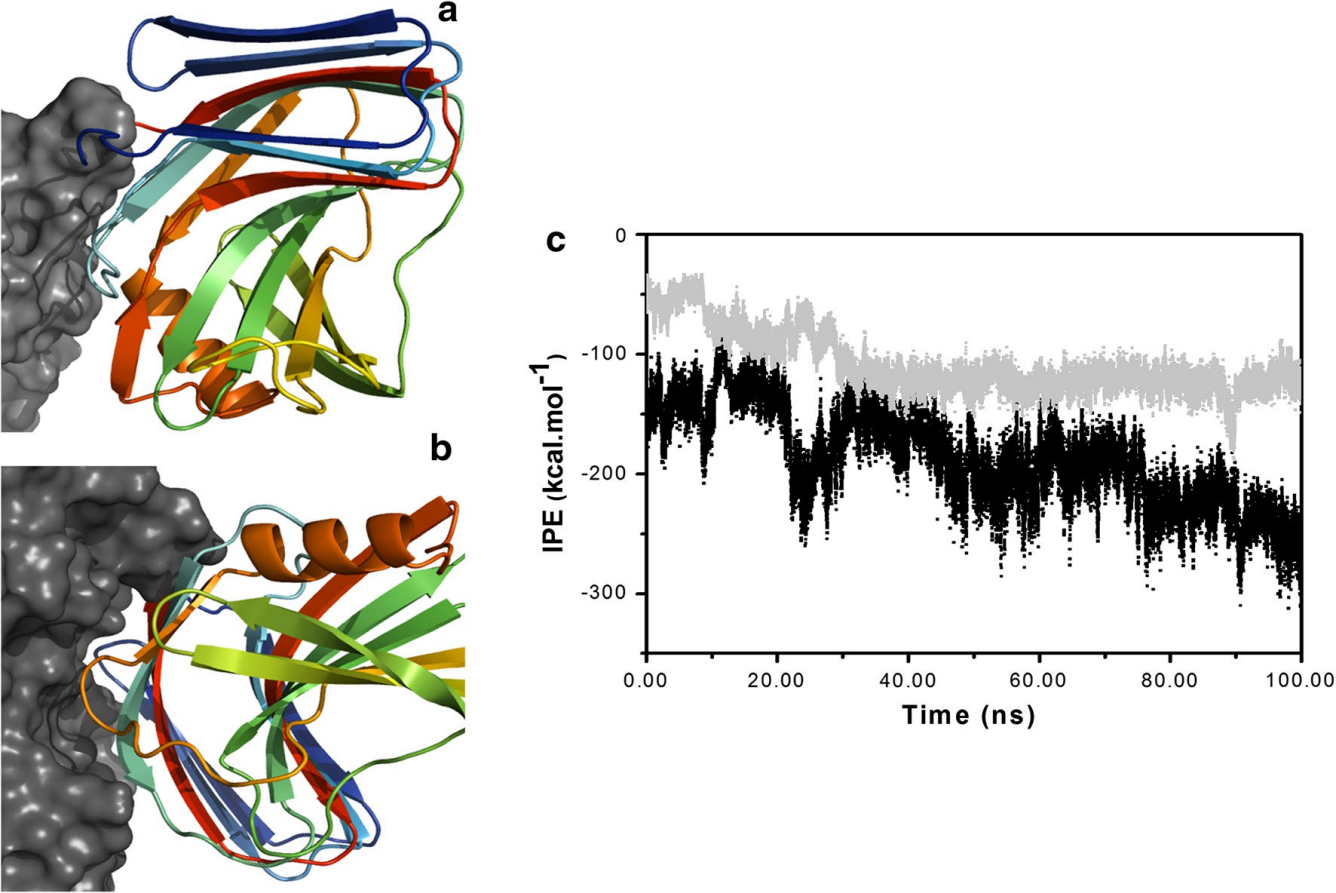
Representations of the final XynA-XBP chimera structures after MD simulations. **a** Chimera 2091A; **b** 2621B. Both *panels* show details of the inter-domain interface, where the XynA domain is shown as a *rainbow ribbon*, and regions from the surface of the xylose-bound XBP domain are shown in *gray*. **c** Comparison of the inter-domain interaction potential energy (IPE) as a function of simulation time for xylose bound 2091A and 2621B chimeras. Short-range potentials (<1.0 nm) between all amino acid residues in the XBP domain and all residues in the xylanase domain are shown for the 2091A (*gray dots*) and the 2621B (*black dots*) chimeras. The structural representations were prepared using the PyMol software [82].

Attempts to crystallize the chimeric proteins were unsuccessful and, therefore, molecular dynamics (MD) simulations were used to gain further insights as to the structural basis for the increase in the catalytic efficiency of the chimeras. The formation of a stable structure during the MD simulations was evaluated by the root mean square deviation (RMSD) for the Cα atoms as a function of time. By this criterion, stability was achieved after ~40 ns for the XBP in both the 2091A and 2621B chimeras, where RMSD values between 0.30 and 0.35 nm were attained. The xylanase domain achieved stability after ~20 ns in both chimeras, with RMSD values between 0.20 and 0.25 nm (Additional file 1: Fig. S6). A visual analysis of the structures generated by the MD simulations revealed the formation of a protein–protein interface between the XBP and XynA domains for both chimera during the first 20 ns and contact which was maintained over the course of the simulations (Fig. 4; Additional file 1: Fig. S7). These inter-domain interfaces were structurally and energetically distinct. In the 2091A chimera, the inter-domain interface was formed by the posterior portion of the palm region of the xylanase while in chimera 262 the xylanase participates in the interface through the fingers region (Fig. 4a, b). The interaction potential energy (IPE) between the two domains as a function of time for both chimeras (Fig. 4c) reveals the energetic difference between the two interfaces. The total average IPE of the protein–protein interactions over the last 100 ns of the MD simulations for the chimeras shows values of −117.0 ± 22.2 kcal mol^−1^ and −188.7 ± 41.4 kcal mol^−1^ for the 2091A and 2621B, respectively. This shows that the 2621B presents a more extensive interface and energetically more favorable protein–protein contacts in comparison to the 2091A.

### Computational alanine-scanning mutagenesis

**Fig. 5:**
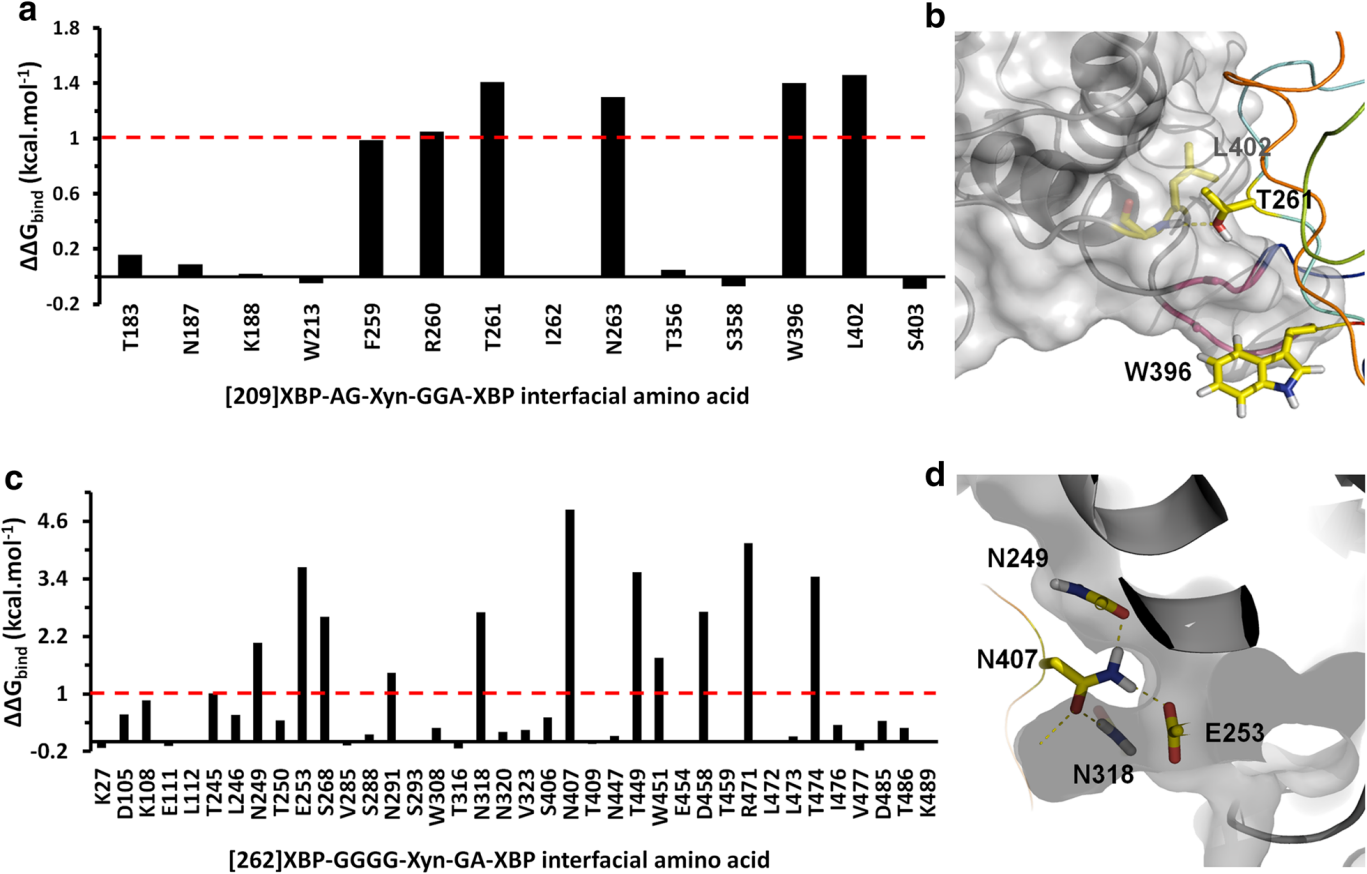
Hot spot residues at the protein–protein interface between XBP and XynA by MD simulations. Changes in binding free energy (ΔΔ*G*_bind_) on alanine mutation of interface residues for **a** 2091A and **c** 2621B. Amino acids with a ΔΔ*G*_bind_ > 1.0 kcal/mol (*red dashed line*) are considered to be critical at the interface. **b** Three hot spot residues (T261, W396 and L402) in the 2091A chimera. T261 and W396 are inserted into a surface cleft of the XBP, and T261 is involved in hydrogen bond formation with a XBP peptide bond (*yellow dashed line*). **d** Four hot spot residues (N249, E253, N318 and N407) of the 2621B chimera. The XynA N407 residue has a large contact area with the XBP, participating in four hydrogen bonds (*yellow*, *dashed lines*). The interfacial residues are shown as stick representation in *yellow*, and the XynA moiety as a ribbon. The XBP is shown as a cartoon and solid surface (in *gray*) and the linker region is in *pink*. The structures representations were prepared using the PyMol software [82].

In these simulations, hot spots at the protein interface were identified by mutating all of the residues contributing the interface of the chimeras were to alanine residues, and an increase in the calculated binding free energy is an indication of the destabilization of the protein–protein interface. The residues that showed binding free energy, ΔΔ*G*_bind_ ≥ 1 kcal mol^−1^, are considered to be those defined as potential hot spot residues [30]. Figure 5 shows the ΔΔ*G*_bind_ for residues together with a structural representation of the primary hot spots identified. In the 2621B chimera the hot spot residues identified showed ΔΔ*G*_bind_ values between 2.0 and 5.0 kcal mol^−1^ while in the 2091A chimera these values are between 1.0 and 2.0 kcal mol^−1^. In the 2091A, six residues were predicted to play a role in the interdomain interactions (Fig. 5a), where each residue makes a similar energetic contribution. The 2621B chimera shows 11 hot spot residues with a predominance of asparagine (36%) and threonine (18%), and it is evident that the protein–protein interface (PPI) is larger in this chimera (Fig. 5c). The simulation scores indicate that T261, W396 and L402 are expected to make a more significant contribution to interface stability, and these three residues are illustrated in Fig. 5b. According to the simulation, T261 in XynA could be involved in hydrogen bond formation with the peptide bond between L402 and S403 of XBP. The L402 residue of XBP appears not to directly interact with XynA, suggesting that the effect of mutation to a methyl side-chain group in alanine may result in an increased solvent accessibility [31]. The small side chains of Gly and Ala in the linker appears to permit a more favorable orientation of the W396 residue. Figure 5d shows four potential hot spot residues for the 2621B chimera (N249, E253, N318 and N407). The N407 residue in the xylanase domain in this chimera is inserted into a surface pocket of the XBP, so as to maximize its interactions with other residues. The majority of the hot spot interactions in this chimera involve hydrogen bonds.

### Conformational changes of the xylanase domain

**Fig. 6:**
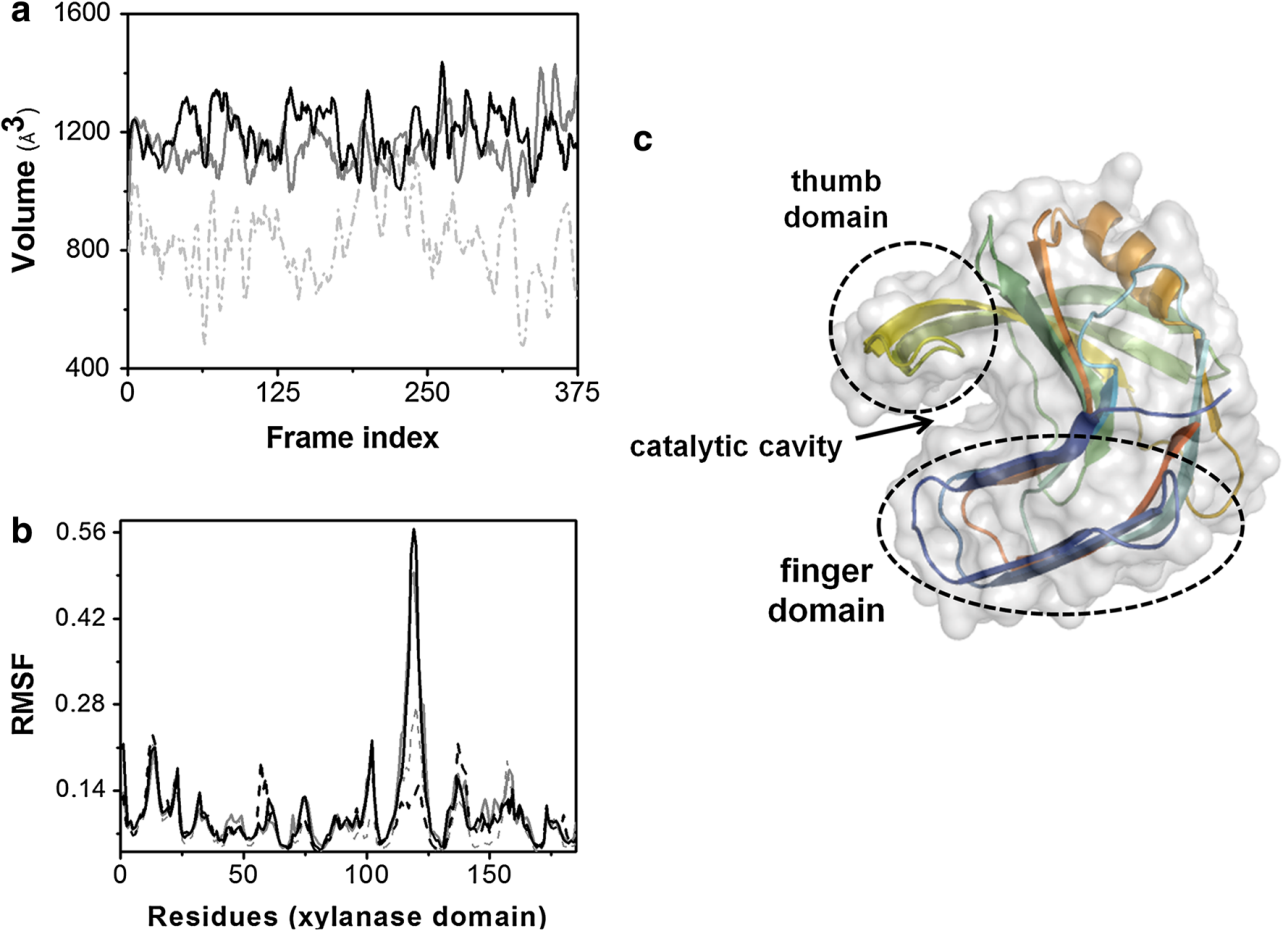
Flexibility and catalytic cavity volume fluctuations in the XynA domain. **a** Time series analysis of the catalytic cavity volume for the XynA domain in the parental XynA (*light gray dashed line*), the 2091A (open, xylose-free, *dark gray line*) and 2621B (open, xylose-free, *black line*). **b** Conformational fluctuations of XynA domain in 2091A (*gray*) and 2621B (*black*) without xylose (*dashed line*) and with xylose (*solid line*). The root mean square fluctuations (RMSF) were calculated for all Cα atoms of all XynA domain residues (residue 1–185) over the final 100 ns. **c** Three-dimensional structure of the XynA highlighting the catalytic cavity located between the palm and fingers domains. Access to the active site cleft is determined by the orientation of the thumb domain. The structure representations were prepared using the PyMol software [82].

In both chimeras, the presence of xylose bound to the specific site of XBP generated an increase in the xylanase activity of the XynA. With the objective of understanding the structural basis of this effect, the volume of the catalytic cavity of the xylanase and the change in the flexibility of the residues in the XynA catalytic domain were calculated from the MD simulations. Figure 6a, b shows the volume of the catalytic cavity during a specific portion of the simulation, represented by the frame index, and the root mean square fluctuation (RMSF) per residue, respectively. The domains and catalytic site of the xylanase are shown in Fig. 6c. The conformational changes that occurred as a consequence of chimer formation were estimated by comparing the volume of the catalytic cavity between the parental xylanase and the xylose-free chimeras over the course of the trajectory (Fig. 6a). The average volumes of the catalytic cavities were 869, 1,202 and 1,157 Å^3^ for XynA, 2091A and 2621B, respectively. The fluctuations of the cavity during the simulations were ±30% for XynA and ±15% for the chimeras. The frames shown in Fig. 6a were selected to illustrate the common behavior of the catalytic cavity volumes of the XynA in the three cases. Between 190 and 250 frames, the XynA showed approximately the same volume as the chimeric enzymes.

The RMSF calculated for each residue (Fig. 6b) clearly shows a large difference in the fluctuation of the positions of the alpha carbons of the thumb domain residues when comparing the XynA domains in the chimeras in the presence and absence of xylose. The main chain in the thumb domain region of the XynA (between residues 110 and 125) in both chimeras in the presence of xylose shows significantly higher flexibility than in the chimeras without xylose. Using the RMSF as a flexibility parameter, the thumb domain of the xylanases in the presence of xylose is approximately two- to fourfold more flexible in the 2091A and 2621B, respectively, as compared to the parental XynA.

## Discussion

The fusion of two or more enzyme amino acid sequences to form a single polypeptide has been widely used as a tool in protein engineering and has been proven to be advantageous for the development of biocatalysts for the treatment and saccharification of biomass [32–36]. The most commonly used approach is the simple fusion of the N-terminus of one enzyme with the C-terminus of the other (so called “end-to-end” fusion), and this domain insertion strategy permits the fusion of two proteins in a wide variety of configurations. Alternatively, one enzyme may be inserted at a specific amino acid position of the other enzyme. Although this limits the number of inter-domain configurations in the case of that specific chimeric protein, when fusion is performed at all amino-acid positions the result is an increase in the inter-domain conformational diversity. This expansion in the number of possible orientations enhances the possibility creating chimeras in which structural changes in one domain affects the activity of the other. However, since the number of possible geometries created by insertion fusion is very large, and the consequences of fusion are not easily predicted, a more practical approach is to evaluate the insertion of one domain into another at multiple points in parallel.

Using this approach, it has previously been demonstrated that the random insertion of a β-lactamase domain into maltose binding protein (MBP) resulted in a series of chimeric proteins in which the β-lactamase activity was modulated by maltose, thereby introducing allosteric behavior into the enzyme [37]. However, random insertion libraries are very large, costly to produce and generally result in a large number of non-functional variants. Furthermore, these libraries show bias towards certain insertion points, and even the largest libraries sample only a small fraction of the immense possibilities in sequence space [38]. A superior strategy is, therefore, to design smaller, high-quality libraries using a semi-rational approach. In the present study, semi-rational domain insertion was applied to an enzyme that degrades lignocellulosic compounds, with the goal of creating a chimera that shows positive modulation by the final product.

The semi-rational domain insertion libraries were constructed in two steps. The first involved the insertion of the xylanase (XynA) into a xylose binding protein (XBP), where the insertion points were chosen based on the structures of a PBPs with homology to XBP that had previously been shown to accept the insertion of a beta-lactamase domain while maintaining the binding capacity for their specific ligands [23]. Of the clones analyzed in this first stage, ~10% showed xylanase activity, which is an improvement when compared to the results of random insertion of beta-lactamase into MBP (maltose binding protein), in which only 0.8% of the clones showed catalytic activity [8]. However, taking into consideration that the ligation efficiency between pSkunk2_XBP and XynA was around 80% and that only 50% of the XynA insertions were in the correct orientation in relation to the XBP reading frame, it was expected that approximately 40% of the clones would have xylanase activity. Thus, the smaller observed value suggests that in roughly three-quarters of the insertion positions, the structure of xylanase was perturbed in such a way as to severely prejudice the catalytic activity of the enzyme. In this first stage, the stimulatory effect of xylose in the allosteric clones was no greater than 1.2-fold, and this may be due to limitations imposed by the relative orientations between the two domains. Previous studies have shown that insertions and deletions are frequently found between the junctions of the domains in proteins with a high switching effect [22, 25, 37, 39, 40]. Therefore, to increase the repertoire of interface contacts between the domains, the inter-domain distance was altered by creating linker libraries based on the two most promising insertion positions (A209-210 and D262-263).

Of the total number of clones analyzed from the linker library at position 209 in the XBP, ~0.05% showed an enhanced activity in the presence of xylose, as compared to the same construction without the linker. For the linker library at position 262, this percentage was 0.6% (12-fold higher in relation to position 209) indicating a greater propensity for the creation of allosteric enzymes at this position. The increase in the switching effect through the addition of linkers between the domains confirms recent studies suggesting that inter-domain linkers play a crucial role in the creation of allosteric protein switches by increasing the inter-domain conformational heterogeneity [41–43]. The optimal temperature for enzymatic activity of the chimeric enzymes was 40 and 50°C for the 2091A and 2621B, respectively, as compared to 45°C for the XynA (Fig. 2b). The fusion of xylanase involves both the N- and C-terminal regions, and the differences in the temperature effect profiles may be the consequence of the known influence of both these regions on the XynA thermostability [44, 45]. The higher temperature maximum of the 2621B may also be explained by the extended protein–protein interface between the XBP and the fingers domain of the xylanase (Fig. 4b), a known structural determinant of thermostability in GH11 xylanases [46]. In this context it is noteworthy that the formation of a protein–carbohydrate interface involving residues in the “fingers” region has recently been shown to stabilize glycosylated forms of the XynA [47].

The kinetic properties of the xylanase activity of the chimeras presented considerable alterations in comparison with the xylanase alone, where a ~threefold increase in the *k*_cat_ values for the chimeras was observed in the presence of xylose (Table 3). A 1.5-fold increase was also observed in the *K*_M_ value for the 2621B. Previous NMR studies on the catalytically inactive XynA E78Q mutant have shown that the binding xylo-oligosaccharides not only at the active site but also at a secondary binding site (SBS) comprised of a surface cleft that is distant from the active site. Furthermore, it was demonstrated that the SBS acts in a cooperative manner with the active site and that mutations in the SBS led to a significant increase in the *K*_M_ value [48]. Alteration of the exposure of the SBS in the XynA due to fusion with XBP could explain the increase in the *K*_M_ value. The chimeric enzymes demonstrated a higher hydrolytic efficiency against the natural substrate in relation to the parental xylanase. However, the stimulatory effect of xylose on sugar cane bagasse hydrolysis was less than with RBB-xylan, and this may be due to the complexity of milled sugarcane bagasse, which contains 21.7% xylan, 22% lignin and 45% glucans [49]. These polymers are interlinked by covalent bonds forming a recalcitrant lignocellulosic matrix [50], with an effective pore size that hinders enzyme access [51]. Thus the larger size of the chimeric enzymes may result in their decreased access to the polysaccharides in the intact bagasse.

Molecular communication between distinct binding sites located in different protein domains in allosteric enzymes may occur through the formation of a protein–protein interface [43, 52]. As a consequence of the approximation between the XynA and XBP domains, the MD simulations suggested such an interface was created in both chimeras, and this offers an explanation for the observed activity modulation by xylose. The 2621B showed a greater effect of xylose, and the predicted protein–protein interface is around 72 kcal/mol more stable than that in the 2091A. Closer analysis indicates that the 262B interface potential energy is dominated by a relatively small number of inter-domain residue contacts, which define “hot spots” at the interface. The more extensive protein/protein interface in 2621B shows a cluster of hot-spot residues and presents complementary surfaces rather than simple contacts between the residues of both proteins. Hot spot residues tend to appear as clusters at interfaces [53]; thus interactions between buried residues become more prominent in comparison with residues on the periphery of the interfacial region [54]. Strong protein–protein interactions are concomitant with the exclusion of water molecules between the binding regions, which promotes lower energies in the hot spot residues [55]. The MD simulation results provide explanations for the structural and energetic basis of the protein–protein interface, and open the possibility that the properties of the chimera may be further optimized by mutation of these hot spot residues.

The MD simulations also revealed that the volume of the catalytic cavity of the parental xylanase is reduced in comparison with those in the chimeras (Fig. 6a). As previously reported [56], opening of the thumb domain leads to greater solvent exposure of the catalytic site and this conformation change is correlated with an increase in the activity of GH11 xylanases. Thus, the increase in the catalytic volumes observed in the current work is consistent with the experimental results. Indeed, the RMSF values suggest that the thumb domain of the xylanase domain in the chimeras is more flexible when xylose is bound to XBP. Since the xylan substrate has direct contact with many residues in this domain [57], increasing the flexibility of the thumb domain residues may improve the conformational adaptation of the substrate in the catalytic site. Thus, MD simulation results suggest that chimera formation has two distinct effects on the xylanase domain: an overall increase in the volume of the catalytic cavity and a xylose dependent increase in the flexibility of the thumb domain.

## Conclusions

The experimental approach used here has been shown to be effective for the creation of a glycosyl-hydrolase that is stimulated by the product of hydrolysis and represents the first time that this concept has been used for engineering an enzyme for the treatment of biomass. The results not only contribute to the understanding of the molecular control mechanisms needed for the modulation of the industrial enzymes used in this sector, but also suggest that this strategy may be explored to create enzymes for various biotechnological applications.

## Methods

### Plasmid construction

The plasmid pSkunk2_XBP was constructed using circular polymerase extension cloning (CPEC) [58]. The region comprised of the signal sequence (Ss) and the xylF gene (XBP) from *Escherichia coli* (Gene ID: 948090) was amplified from the vector pT7T3GFP_XBP [59] with primers XBPf (5′-ggaggaaggatccatggcatgaaaataaagaacattctactcaccctttgcacc-3′) and XBPr (5′-ccctgaggttactagtttacagctcgctctctttgtggaatccg-3′). pSkunk2 is a 3.2-kb phagemid derived from pDIM-C8 [60], in which the Kanamycin resistance marker is substituted with the streptomycin/spectinomycin (Sm/Spec) resistance. This vector was amplified with primers pSkunk2f (5′-cggattccacaaagagagcgagctgtaaactagtaacctcagggttatgtatgcacaagg-3′) and pSkunk2r (5′-agtagaatgttctttattttcatgccatggatccttcctcctgtgtgaaattgttatcc-3′). The overlapping regions of the two sets of primers are underlined in the sequence. An equimolar mixture of the two amplified fragments (PS-XBP and pSkunk1) was submitted to the CPEC reaction and used to transform *E. coli* DH5α cells as described previously [58], and the correct pSkunk2_XBP construct was confirmed by nucleotide sequencing. The xynA gene from *Bacillus subtilis* (GeneID: 939861) was cloned into the plasmid pT7T3 18U (2883pb—Amersham Pharmacia), as described previously [36], generating the construct pT7T3/XynA.

### Library creation by semirational insertion of XynA into XBP

Fragments corresponding to the complete vector pSkunk2_XBP (4,181 bp) were generated by multiplex inverse PCR [61] starting from specific codons of XBP. Based on previous results with ribose binding protein (RBP) and glucose binding protein (GBP), 144 codons within the sequence of XBP were selected, and 144 primer pairs with melting temperatures (Tm) close to 60°C. The PCRs were performed in the following conditions: 3 ng of pSkunk2_XBP, 3% DMSO, 1.1 M betaine, 50 nM oligonucleotides (forward and reverse) and 1X Phusion^®^ High-Fidelity Master Mix (NEB) with sterile water to a final reaction volume of 20 μL. The amplifications were performed in a thermocylcer programmed to generate 98°C for 30 s followed by 30 cycles of: 98°C for 10 s, 60°C for 20 s and 72°C for 130 s. After 30 cycles, a final step at 72°C was performed for 5 min. The PCR products were mixed and applied to a 0.8% agarose gel in TE buffer, and the 4,181-bp fragment was gel purified. The xynA gene was PCR amplified without a stop codon from the pT7T3/XynA vector using phosphorylated primers, and ligated to the gel purified pSkunk2_XBP plasmid. The ligation reaction contained ~1 µg of plasmid with a molar ratio of 5:1 insert:plasmid and 1,000 U/µg of T4 DNA ligase (NEB), 1× ligase buffer, and 5% PEG8000. The reaction was incubated at 22°C for 14 h, purified using a DNA Clean and Concentrator™ column (Zymo Research), and eluted with 100 µL of water/column. The samples from the ligations were concentrated and used to transform electrocompetent kanamycin resistant JW3538-1 *E. coli* cells carrying the XBP gene (xylF) knockout (strain from the Coli Genetic Stock Center, USA). After incubation and regeneration for 1 h/37°C, the cells were plated on LB-agar containing 34 µg/mL kanamycin and 50 µg/mL streptomycin, on bioassay plates (245 × 245 × 25 cm). After growth, colonies were harvested in storage media [LB + 50% glycerol (vol/vol)] and stored in cryotubes at −80°C.

### Construction of the linker library

The clones from the pSkunk2_XBP library with the xylanase inserted between Ala 209 and residue 210 of XBP (A209-210) and between Gln 262 and residue 263 of XBP (Q262-263) selected presented the xylose activation effect (see next section) and were used for the construction of a linker library. Primer pairs were designed to insert random combinations of 0-4 glycine and/or alanine residues at the junctions between the XynA and XBP coding regions. For the complete randomization of the linkers, 0–4 repetitions of the codons GSC or GSG (S represents a guanine or a cytosine) were added to the 5′ and 3′ ends of the primers (Additional file 2: Table S1), and an equimolar mixture of the phosphorylated primers was used to amplify the xynA by PCR from the pT7T3/XynA vector. In parallel, two PCR reactions were performed to amplify and linearize the pSkunk2_XBP plasmid at the two selected positions (see Additional file 2: Table S1 for the two pairs of primers used). The amplification products (xylanase gene: ~555 bp and linearized plasmid: 4,181 bp) were gel purified and ligated, and used to transform *E. coli* JW3538-1 ΔxylF cells. After plating on LB-agar containing 34 µg/mL kanamycin and 50 µg/mL streptomycin, in bioassay plates (245 × 245 × 25 cm), the colonies were harvested in storage media [LB + 50% glycerol (vol/vol)] and stored in cryotubes at −80°C.

### Screening for the catalytic activity of xylanase (XynA)

A 10 µL aliquot of cells from the libraries stored at −80°C was plated on LB media containing 34 µg/mL kanamycin and 30 µg/mL streptomycin. After incubation at 37°C for 16 h, individual colonies were transferred to 384-well micro plates containing 60 µL selective tryptone broth (TB) (per liter, 10 g of tryptone and 5 g NaCl) using an automated colony picker (Kbiosystems-K6). The plates were incubated at 37°C/24 h and replicated on bioassay plates (245 mm × 245 mm × 25 mm) containing TB-agar media, supplemented with 0.6% (m/v) xylan, 1% (m/v) xylose; 34 µg/mL kanamycin; 30 µg/mL streptomycin and 1 mM IPTG. For linker libraries, cells were replicated on bioassay plates (245 mm × 245 mm × 25 mm) containing TB-agar media, supplemented with 0.02% (m/v) RBB-xylan (Remazol Brillian Blue Xylan) (Sigma), 34 µg/mL kanamycin, 30 µg/mL streptomycin and 1 mM IPTG, with and without 1% (m/v) xylose. After incubation of the plates at 37°C for 24 h, the clones expressing xylanase in the semirational insertion library of xylanase into XBP were located using the formation of pale halos after staining with Congo Red [62]. The clones expressing xylanase in the linker libraries were identified by the formation of halos around the colonies, resulting from the degradation of the RBB-xylan. In both libraries, those colonies presenting clearer halos on the plates with xylose were selected and were denominated as XynA+ clones.

### Measurement of xylose stimulated catalytic activity

The XynA+ clones were grown in TB supplemented with 34 µg/mL kanamycin, 50 µg/mL streptomycin and 0.5 mM IPTG for 48 h in 96-well plates (deep well). The supernatants were analyzed for hydrolysis of RBB-xylan (Remazol Brilliant Blue Xylan, Sigma), using a modification of the protocol developed by Biely et al. [63]. In this protocol, 50 µL of supernatants was mixed with 50 µL of a solution containing RBB-xylan (4 mg/mL), in 100 mM acetate buffer (pH 5.5), in the presence or absence of 1% (m/v) d-xylose (Sigma), and incubated at 37°C for 12 h. After incubation, the reaction was stopped by the addition of two volumes (200 µL) of 96% (v/v) ethanol. The insoluble material was removed by centrifugation (2,000*g*/2 min), and the increase in the absorbance of the supernatant was monitored at 595 nm. The clones that showed the highest activity in the presence of d-xylose compared to the absence of d-xylose were selected.

### Expression and purification of the recombinant enzymes

The XynA and the chimeric enzymes were expressed in *E. coli* [Rosetta™ (DE3)] transformed with pET28a (+) (Novagen) carrying XynA or 2091A or 2621B grown in HDM medium containing (per liter) 25 g of yeast extract, 15 g of tryptone, 1.2 g of MgSO4, supplemented with 34 µg/mL kanamycin and 40 µg/mL chloramphenicol. The cells were grown at 37°C to OD_600_ of 0.6, and expression was induced with 1 mM isopropyl-d-thiogalactopyranoside for 4 h. Cells were harvested by centrifugation (8,000*g*, 4°C, 10 min). Whole-cell extracts were prepared from cell pellets by ultrasonication in 4% (v/v) of the original culture volume of lysis buffer (100 m M HEPES (pH 7.5), 300 mM NaCl, 0.5 mM phenylmethylsulfonyl fluoride, 1% (v/v) Triton X-100, and 20 mM imidazole). The cell extracts were cooled on ice and cleared of cell debris by centrifugation (10,000*g*, 4°C, 30 min). The supernatants were loaded on an immobilized metal affinity column Ni–NTA (Amersham Biosciences) pre-equilibrated with a buffer containing 100 mM HEPES, 300 mM NaCl, and 20 mM imidazole (pH 7.5). The column was washed with buffer containing 100 mM HEPES (pH 7.5), 300 mM NaCl, and 40 mM imidazole until no further reduction in the *A*_280_ was observed. Protein was eluted with 300 mM imidazole, and protein samples were dialyzed against 20 mM Tris–HCl (pH 8.0) and 200 mM NaCl and stored at 4°C for future use. The protein concentrations were determined by measurement of the *A*_280_.

### Enzyme activity assays

The effect of pH on xylan hydrolysis by the purified enzymes was determined at 40°C in 50 mM with 0.2% (w/v) RBB-xylan substrate (Sigma) buffered with acetic acid/acetate (pH 4.5–5.5), potassium phosphate (pH 5.5–6.5), MOPS-NaOH (pH 6.5–7.5) and Arginine-NaOH (pH 9.0). The effect of temperature on xylanase activity was conducted at temperatures between 30 and 55°C in 50 mM acetate, pH 5.5. The xylanase kinetic parameters were determined using the RBB-xylan substrate in concentrations ranging from 0.5 to 10 mg/mL, with and without 1% (w/v) d-xylose (Sigma). The reactions were initiated by the addition of 20 nM of the purified enzyme to acetate buffer (pH 5.5) at 37°C. After 15 min, the enzyme was inactivated by incubation at 80°C for 10 min, followed by incubation at 4°C for 5 min. Five hundred microliter of ethanol was then added and the mixture incubated at 25°C for 15 min. The samples were centrifuged at 13,200 rpm for 2 min and 580 µL of each sample were transferred to a 1-cm cuvette for measurement of the absorbance at 595 nm [64]. The absorbance values were converted to μmols of released dye (*ε* = 8,266 M^−1^ cm^−1^) [65] to determine the catalytic constants. All enzymatic activities were determined in triplicate, and the maximum velocity (*V*_max_), Michaelis constant (*K*_M_), and catalytic constant (*k*_cat_) were calculated by nonlinear regression fitting of the data to the semi-logarithmic form of the Hill equation using the SigrafW software [66].

### Determination of the equilibrium dissociation constant with xylose

XBP and chimeric enzymes at a concentration of 3 μM were titrated with xylose (Sigma–Aldrich, St. Louis, MO, USA) over the concentration range 0–1 μM in buffer containing 20 mM Hepes (pH 7.5). The changes in fluorescence emission were measured with a Hitachi F-4500 spectrofluorimeter at 25°C, using a stirred 1 cm optical path length quartz cuvette. Excitation and emission wavelengths were set to 295 and 310–345 nm, respectively. The equilibrium dissociation constant (*K*_d_) for each xylose/protein complex was estimated by nonlinear curve fitting with a sigmoidal dose–response function using the OriginPro 8 software (OriginLab Corporation, Northampton, MA, USA).

### Enzyme assays using natural substrate

Milled sugarcane bagasse (particle size 0.2 mm) was treated with 80% ethanol and washed thoroughly with 50 mM phosphate buffer (pH 6.0) to remove residual soluble sugars. A 1% w/v suspension of the treated and washed substrate was prepared in the same buffer and mixed with either 25 nmol of purified chimeras, with 25 nmol of individual purified xylanase or with an equimolar mixture of 25 nmol of xylanase and 25 nmol of XBP, in a final reaction volume of 50 mL. The reaction was incubated at 37°C for 2 h in a shaker at 200 rpm to avoid substrate precipitation, and the total reducing sugar release was measured by the DNS method as described previously [35].

### Modeling and molecular dynamics simulations

Initial atomic coordinates of the chimeras were obtained from the structures of xylanase from *B. subtilis* (PDB code 1XXN [56]) and XBP from *E. coli* (PDB code 3M9W for open xylose-free and 3MA0 for closed xylose-bound [67]) as templates for building the structural models of the chimeras by comparative modeling techniques with the program MODELLER 9.13 [68]. The structural models were validated utilizing the program Procheck [69]. Initial 3D model of the chimeras were submitted to an energy minimization step using the steepest descent algorithm [70]. Subsequently, each chimera was solvated with SPC water molecules [71] at a concentration of approximately 53.0 mol/L in dodecahedral simulation boxes. The protonation state of the ionizable residues at pH7 was determined by Poisson-Boltzmann based pKa calculations using the H++ program [72]. Three sodium ions were inserted into the simulation boxes at the most electrostatically favorable positions to ensure the electroneutrality of the systems. Systems were equilibrated for approximately 400 ps by position restrained MD at 300K to eliminate remaining repulsive energies. All systems were simulated in the NVT ensemble at pH 7.0 and 300K, in which the temperature was controlled using the V-rescale thermostat [73]. LINCS [74] and SETTLE [75] algorithms were used to restrain the covalent bonds involving hydrogen atoms in protein and water molecules, respectively. The leap-frog integration algorithm [76] was employed to solve the Newton’s equations of motion with a time step of 2.0 fs. The initial velocities were obtained from Maxwell–Boltzmann distribution at 300K. The long-range interactions were treated using particle-mesh Ewald sum (PME) method [77], with a cutoff equal to 1.2 nm and updated every 10 time-step intervals. The total time of each simulation was determined based on the root mean square deviation (RMSD) behavior in time. Approximately 100 ns were achieved in each MD simulation. All MD runs and analyses were performed with the GROMACS 4.6 toolkit [78] using the GROMOS-96(53A6) force field [79]. The Interaction Potential Energies (IPE) were calculated as the sum of all interaction energies (*E*_*ij*_) between all atoms from Protein A (*i*) and all atoms from Protein B (*j*) according to the following equation:

$${\text{IPE}} = \sum\limits_{i}^{\text{NA}} {\sum\limits_{i}^{\text{NB}} {E_{i,j} } } ,$$

where NA and NB are the total number of protein A and B atoms, respectively. Computational alanine scanning was performed by ROBETTA software [80] to identify the energetically important residues at the protein–protein interface. The volume of xylose binding cavity of xylanase was monitored along the MD trajectories with the Eyrisch and Helms tool [81] EPOSBP (http://gepard.bioinformatik.uni-saarland.de/software/epos-bp). The default parameters of EPOSBP were employed for the measurement of cavity properties such as volume and the residues lining the cavities. Due the large dimension of the target cavity and its dynamic motion during the simulations, the cavities detected were divided into more convenient subcavities, and the reported volumes of the target cavity is the sum of all subcavities defined by the residues lining the substrate binding cavity of xylanase.

## Additional files

Additional file 1: Screening of the effect of xylose on the xylanase activity of the 225 XynA+clones. The xylanase activity in culture supernatants of each clone was measured in the presence and absence of xylose, and the activity ratio in the presence (+ xylose) as compared to the absence (−xylose) was calculated. A ratio (+xylose/−xylose) of 1.0 indicates no difference in the activity in the presence of xylose. Of the 225 clones, 69% (155 clones) showed lower activity in the presence of xylose and 4% (10 clones) showed an increased activity greater than 10% in the presence of xylose. See “Methods” section for further experimental details. Additional file 2:
**Table S1.** Oligonucleotides used for the construction of the linker libraries at XBP positions 209 and 262.

## Abbreviations

GH11: glycosyl Hydrolase family 11
2091A: chimeric enzyme in which the xylanase plus linkers (alanine-glycine in N-terminus and glycine–glycine-alanine in C-terminus) was inserted after residue 209 of the XBP
2621B: chimeric enzyme in which the xylanase plus linkers (glycine–glycine–glycine–glycine in N-terminus and glycine-alanine in C-terminus) was inserted after residue 262 of the XBP
pSkunk2: vector used in this work
DNS: 3,5-dinitrosalicylic acid
PDB: protein data base
IPTG: isopropyl β-d-1-thiogalactopyranoside
MD: molecular dynamics
