# Supplementary material for: Insertion of a xylanase in xylose binding protein results in a xylose-stimulated xylanase
Source: Biotechnol Biofuels. 2015 Aug 15;8:118. doi: 10.1186/s13068-015-0293-0 (PMC4536891; doi:10.1186/s13068-015-0293-0)
Supplement: Additional file 1: — Screening of the effect of xylose on the xylanase activity of the 225 XynA+clones. The xylanase activity in culture supernatants of each clone was measured in the presence and absence of xylose, and the activity ratio in the presence (+ xylose) as compared to the absence (−xylose) was calculated. A ratio (+xylose/−xylose) of 1.0 indicates no difference in the activity in the presence of xylose. Of the 225 clones, 69% (155 clones) showed lower activity in the presence of xylose and 4% (10 clones) showed an increased activity greater than 10% in the presence of xylose. See “Methods” section for further experimental details. [file 13068_2015_293_MOESM1_ESM.docx]

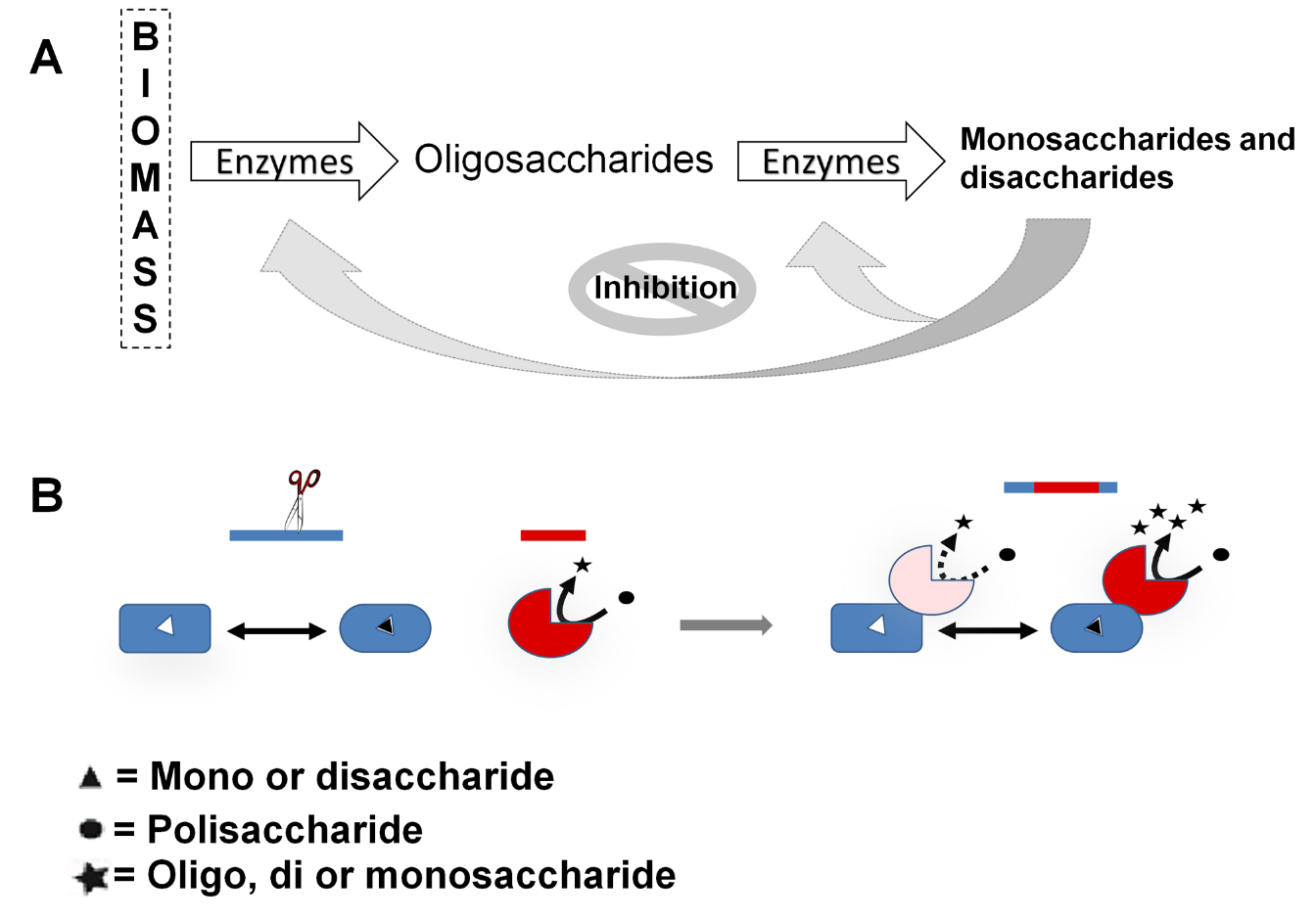


Figure S1: Modulation of enzymatic activity by a reaction product. A) Feedback inhibition by the final product. For many enzymes involved in the degradation of biomass, the accumulation of the final product during the hydrolysis process inhibits the enzymes that catalyze previous reactions. B) The creation of enzymes stimulated by the final product. The desired enzyme is derived from the insertion of a catalytic domain (red) into a binding protein (blue). The binding of the final product (e.g. a monosaccharide) to the binding domain results in an increase in activity of the catalytic domain.


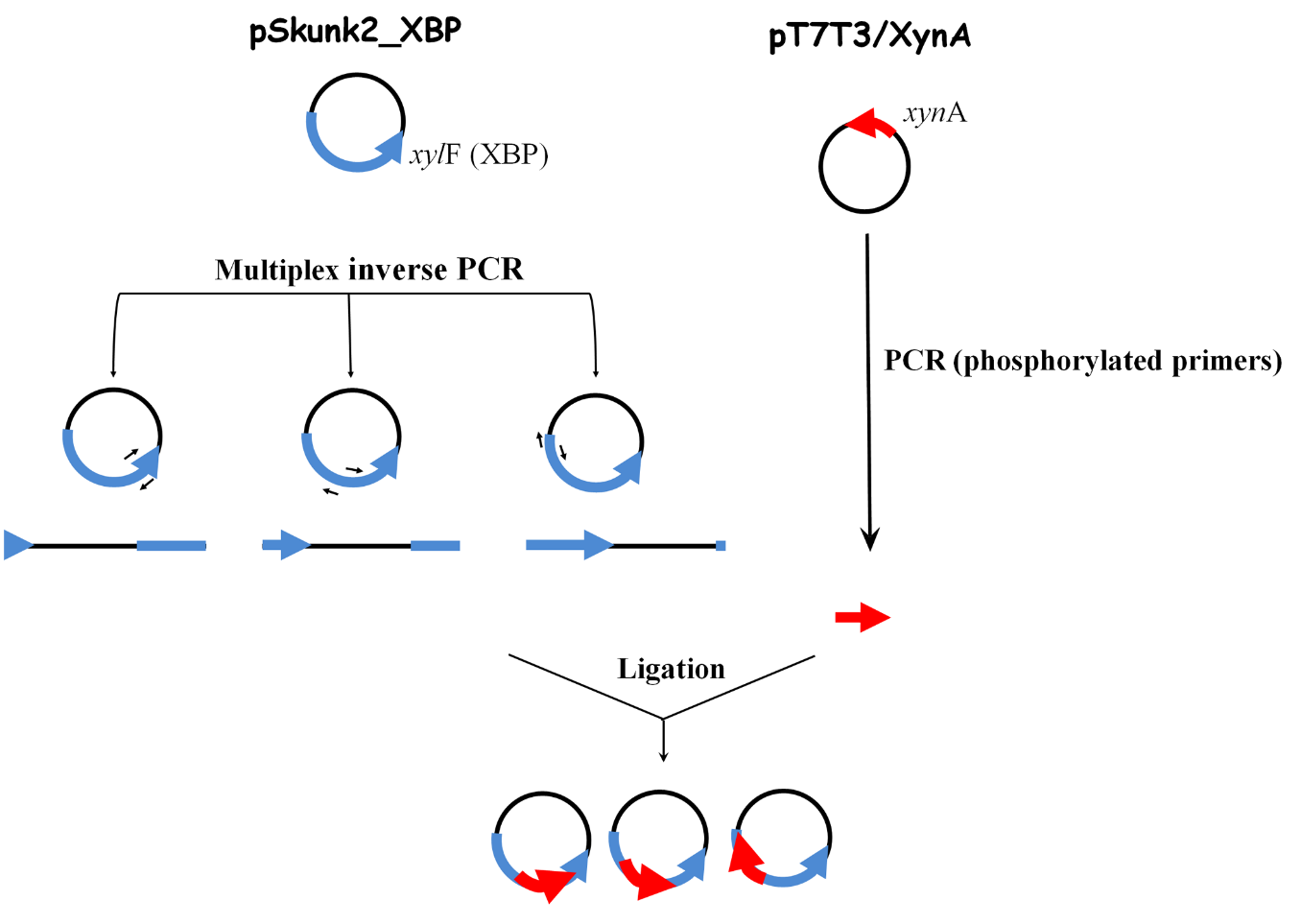


Figure 2. Library construction by semi-rational insertion of XynA into XBP. Schematic representation of the construction of the semi-rational insertion library xylanase-XBP chimeras. The fragment of the *xyn*A gene that encodes the mature xylanase protein (without a Stop codon) is amplified with phosphorylated primers and inserted into the linearized pSkunk2_XBP plasmid at the pre-determined positions.


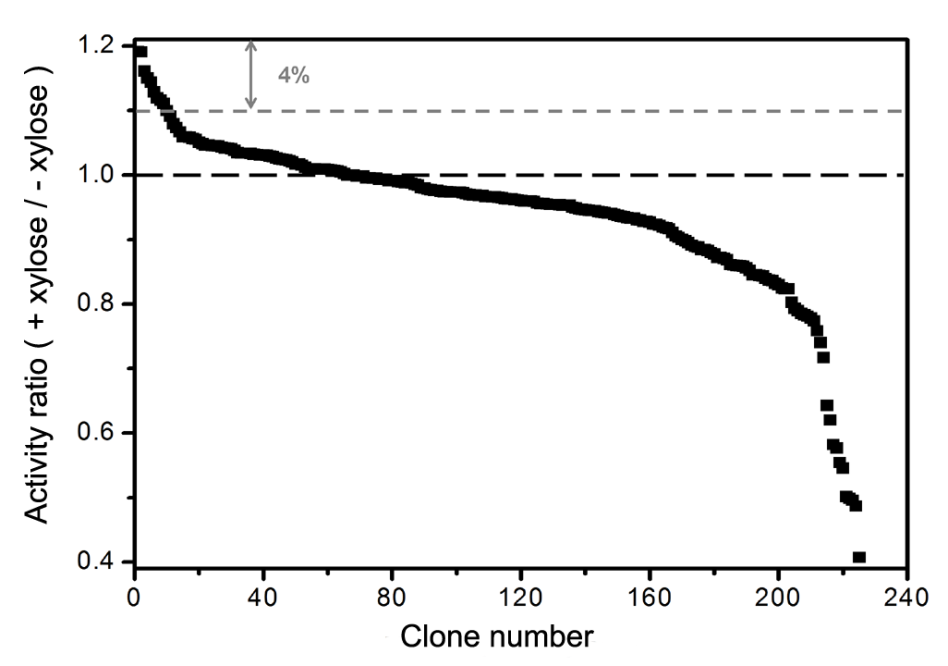


**Figure S3. Screening fot the effect of xylose on the xylanase activity of 225 XynA+ clones.** The xylanase activity in culture supernatants of each clone was assayed in the presence and absence of xylose, and the activity ratio in the presence (+ xylose) as compared to the absence (- xylose) was calculated. A ratio (+xylose/-xylose) of 1.0 indicates no difference in the activity in the presence of xylose. Of the 225 clones, 69% (155 clones) showed lower activity in the presence of xylose and 4% (10 clones) showed an increased activity greater than 10% in the presence of xylose. See materials and methods section for further experimental details.

**
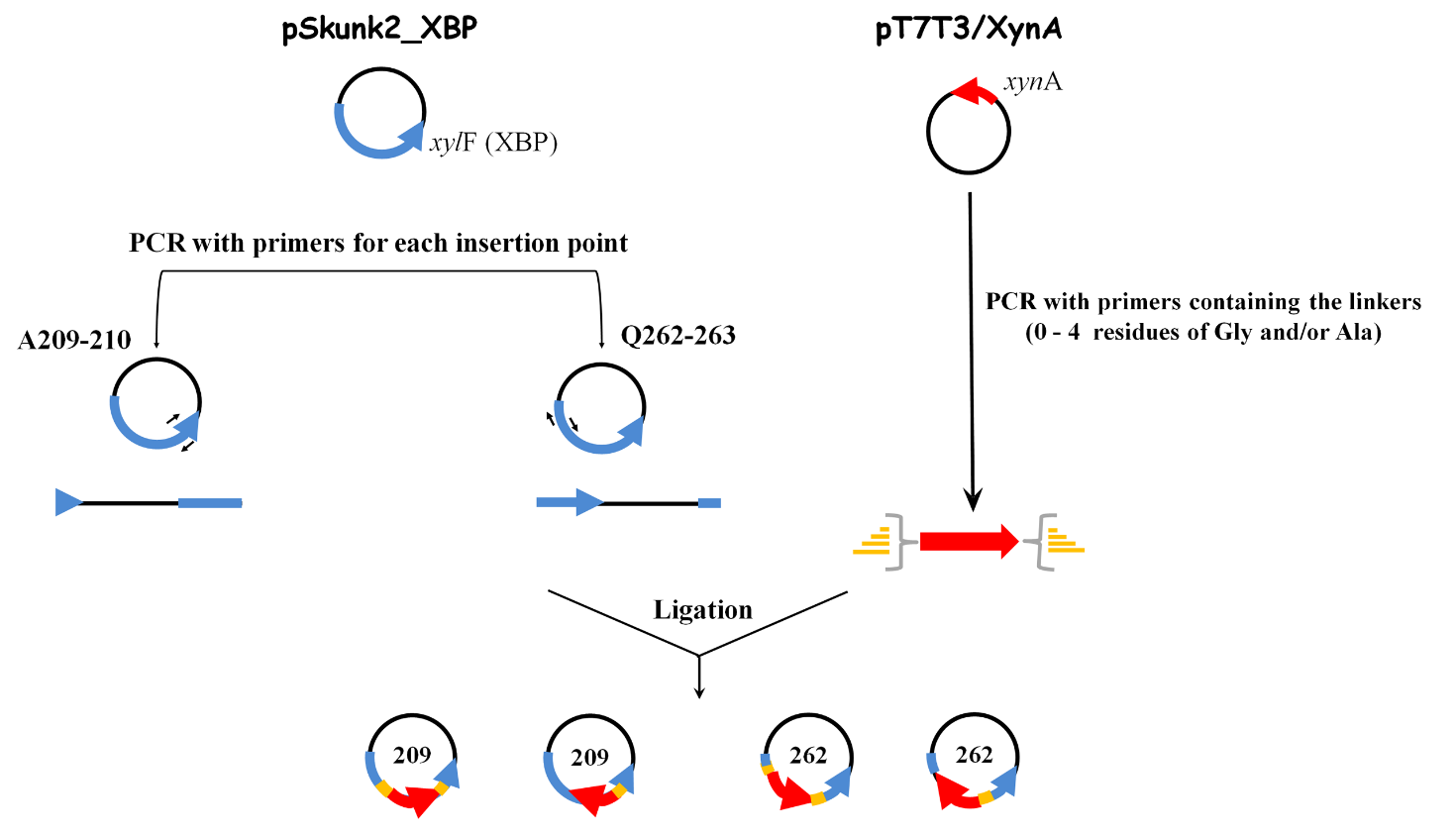
**

**Figure S4. Schematic representation of the inter-domain linker library construction.** The nucleotide coding sequence of the mature XynA protein (without the Stop codon) was amplified with primers that randomly included 0 to 4 codons encoding glycine and/or alanine residues, and the amplification product was ligated to the pSkunk2_XBP plasmid linearized at XBP residue position 209 or 262.


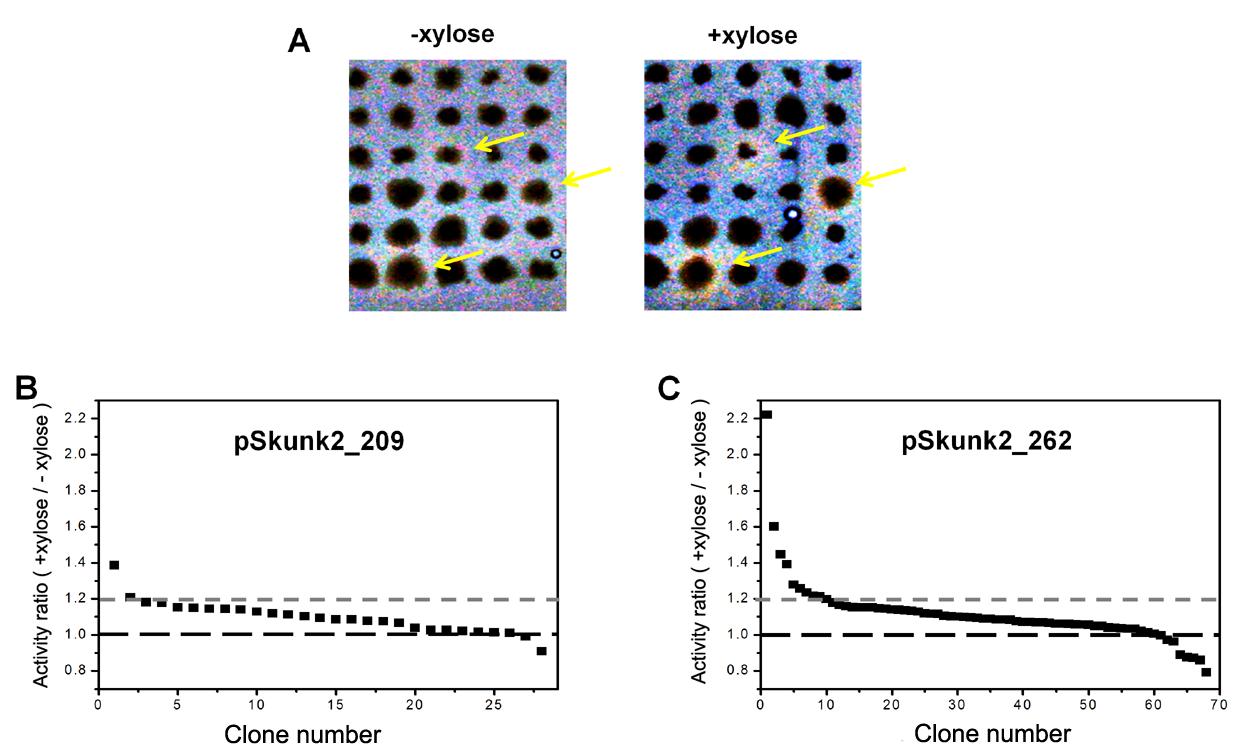


**Figure S5.** (A) Clone selection from the pSkunk2_262 library. Example of halo formation on solid agar plates in the absence of xylose (-xylose) as compared to plates with xylose (+xylose). The arrows indicate colonies which show increased activity in the presence of xylose. The contrast of the image has been enhanced in order to facilitate the visualization of the positive clones. The xylanase activity in the culture supernatants from clones that showed xylose stimulation are shown for; (B) the pSkunk2_209 library, where only a single clone showed an increased activity above 20% in the presence of xylose (short dotted line), and (C0 analysis of the pSkunk2_262 library, where 7 clones showed stimulation of catalytic activity greater than 20%. See materials and methods section for further experimental details.


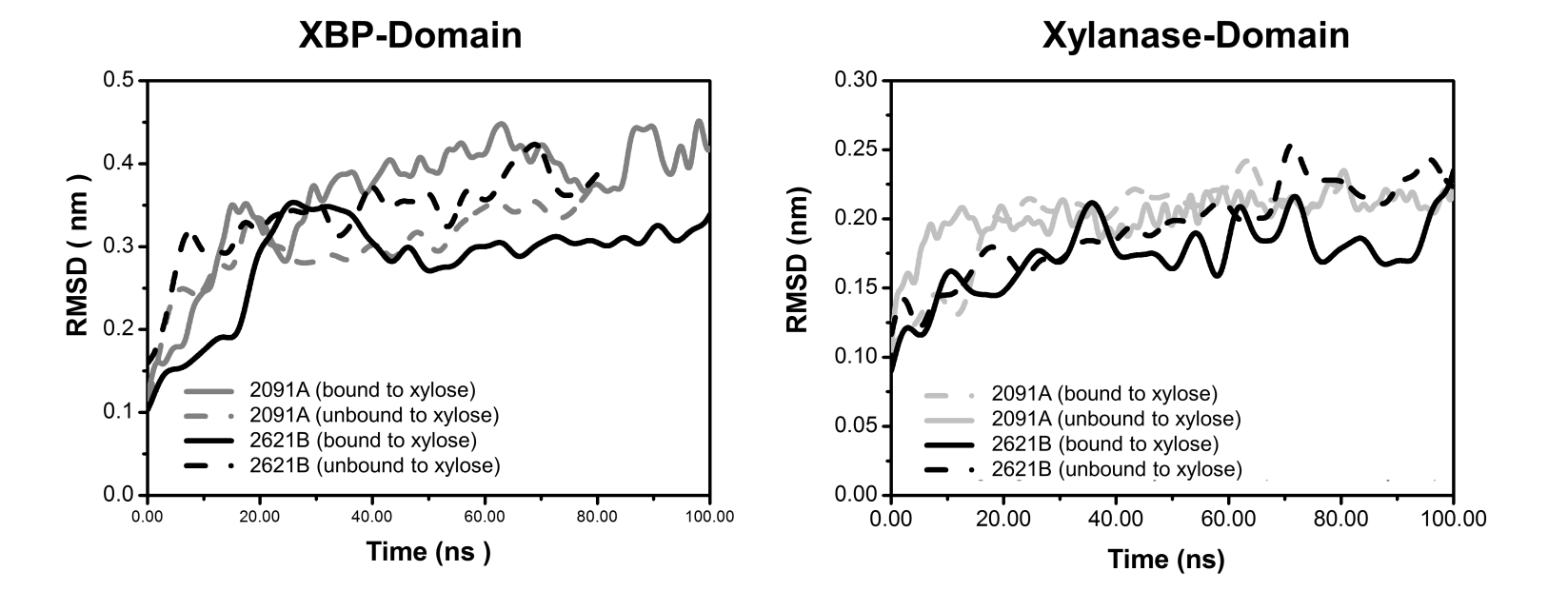


**Figure S6. Root mean square deviation (RMSD) values for the 2091A and 2621B chimeras from molecular dynamics simulations**. The RMSD values of the XBP (left-hand panel) and xylanase (right-hand panel) domains are shown for the 2091A (light grey lines) and the 2621B (black lines), with xylose bound to the XBP domain (solid lines) or without xylose bound to the XBP (dashed lines). See materials and methods section for further experimental details.


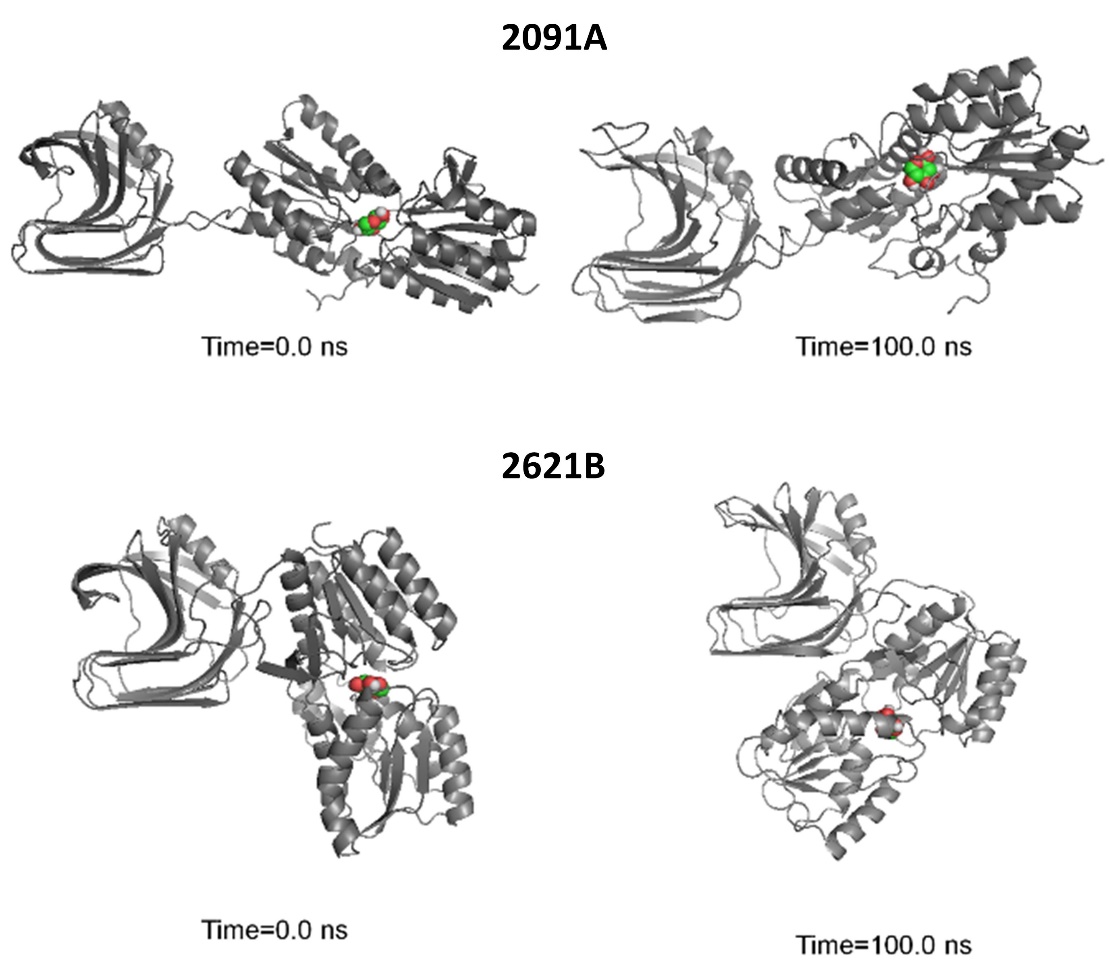


**Figure S7. Structures of the chimeras from the molecular dynamics simulations.** The formation of a protein-protein interface between the XBP and XynA domains is shown for the 2091A (upper panel) and 2621B (lower panel) chimeras. The initial (t = 0 ns) and final (t = 100 ns) structures of each chimera are shown.
