# Supplementary material for: Insertion of a xylanase in xylose binding protein results in a xylose-stimulated xylanase
Source: Biotechnol Biofuels. 2015 Aug 15;8:118. doi: 10.1186/s13068-015-0293-0 (PMC4536891; doi:10.1186/s13068-015-0293-0)
Supplement: Additional file 2: — Table S1. Oligonucleotides used for the construction of the linker libraries at XBP positions 209 and 262. [file 13068_2015_293_MOESM2_ESM.docx]

Table S1. Oligonucleotides used for the construction of the linker libraries at XBP positions 209 and 262.

| Primer name | Sequence |
| --- | --- |
| N-ter xyn0 | GCTAGCACAGACTACTGG |
| C-ter xyn0 | CCACACTGTTACGTTAGA |
| N-ter xyn1 | GSGGCTAGCACAGACTACTGG |
| C-ter xyn1 | GSCCCACACTGTTACGTTAGA |
| N-ter xyn2 | GSGGSGGCTAGCACAGACTACTGG |
| C-ter xyn2 | GSCGSCCCACACTGTTACGTTAGA |
| N-ter xyn3 | GSCGSGGSGGCTAGCACAGACTACTGG |
| C-ter xyn3 | GSCGSCGSCCCACACTGTTACGTTAGA |
| N-ter xyn4 | GSCGSCGSGGSGGCTAGCACAGACTACTGG |
| C-ter xyn4 | GSCGSCGSCGSCCCACACTGTTACGTTAGA |
| XBP209F | CGCGCTTAATGCCTGAATTG |
| XBP209R | CAAGGTTTATCAGGGAAAGT |
| XBP262F | GAACCAAAAGCAGATACCAC |
| XBP262R | CTGACCATTGCCCAACTCAA |
